# The effects of arbuscular mycorrhizal fungi and essential oil on soil microbial community and N-related enzymes during the fungal early colonization phase

**DOI:** 10.3934/microbiol.2017.4.938

**Published:** 2017-12-04

**Authors:** George P. Stamou, Sotiris Konstadinou, Nikolaos Monokrousos, Anna Mastrogianni, Michalis Orfanoudakis, Christos Hassiotis, Urania Menkissoglu-Spiroudi, Despoina Vokou, Efimia M. Papatheodorou

**Affiliations:** 1 School of Economics, Business Administration and Legal Studies, International Hellenic University, 57001 Thessaloniki, Greece; 2 Department of Ecology, School of Biology, AUTH, 54124 Thessaloniki, Greece; 3 Department of Soil Science of Athens, Institute of Soil and Water Resources, Hellenic Agricultural Organization-Demeter, 14123 Lykovrisi, Greece; 4 Department of Forestry and Management of the Environment and Natural Resources, Democritus University of Thrace, 68200 Orestiada, Greece; 5 Department of Natural Environment and Forestry, Technical University of Larissa, 43100 Karditsa, Greece; 6 Laboratory of Pesticide Science, School of Agriculture, AUTH, 54124 Thessaloniki, Greece

**Keywords:** *Rhizophagous irregularis*, *Mentha spicata*, arylamidase, acid phosphatase, asparaginase, glutaminase

## Abstract

The arbuscular mycorrhizal fungi (AMF) and the essential oils are both agents of sustainable agriculture, and their independent effects on the community of free-living soil microbes have been explored. In a tomato pot experiment, conducted in a sandy loam mixture, we examined the independent and joint effects of inoculation with the fungus *Rhizophagous irregularis* and the addition of *Mentha spicata* essential oil on the structure of the soil microbial community and the activity of soil enzymes involved in the N-cycle, during the pre-symbiosis phase. Plants were grown for 60 days and were inoculated with *R. irregularis.* Then pots were treated with essential oil (OIL) weekly for a period of a month. Two experimental series were run. The first targeted to examine the effect of inoculation on the microbial community structure by the phospholipid fatty acids analysis (PLFAs), and enzyme activity, and the second to examine the effects of inoculation and essential oil addition on the same variables, under the hypothesis that the joint effect of the two agents would be synergistic, resulting in higher microbial biomass compared to values recorded in singly treated pots. In the AMF pots, N-degrading enzyme activity was dominated by the activity of urease while in the non-inoculated ones by the activities of arylamidase and glutaminase. Higher microbial biomass was found in singly-treated pots (137 and 174% higher in AMF and OIL pots, respectively) compared with pots subjected to both treatments. In these latter pots, higher activity of asparaginase (202 and 162% higher compared to AMF and OIL pots, respectively) and glutaminase (288 and 233% higher compared to AMF and OIL pots, respectively) was found compared to singly-treated ones. Soil microbial biomasses and enzyme activity were negatively associated across all treatments. Moreover, different community composition was detected in pots only inoculated and pots treated only with oil. We concluded that the two treatments produced diverging than synergistic effects on the microbial community composition whereas their joint effect on the activity of asparaginase and glutaminase were synergistic.

## 1. Introduction

The involvement of a large number of vascular plants in symbiotic relationships with fungi in diverse habitats, indicates the importance of fungi for the plant soil complex. The colonization of plants by the arbuscular mycorrhizal fungi (AMF) affects the quality and the quantity of the host plant root exudates, the structure of the microbial communities in the rhizospheres and the chemotactic responses of specific bacteria [1],[2]. Moreover, interesting interactions are developed between AM fungi and soil bacteria, including the binding of the latter to the fungal spores, the production of volatiles by free living bacteria, and the degradation of fungal cellular walls [3]. On the other side, specialized “mycorrhiza helper bacteria” promote the activity and development of AM fungi with their effects being fungal specific. In particular, the AM fungus *Glomus intraradices* renamed *Rhizophagous irregularis* belonging to the Phylum Glomeromycota has been found in symbiosis with the majority of terrestrial vascular plants [4],[5],[6]. Inoculation with this fungus significantly influenced the development of the bacterial community of tomato rhizosphere [7] due to the suppression or stimulation of one or more susceptible microbial populations [8]. Due to their interactions with members of the rhizosphere microbial community, AM fungi have been used as biofertlizers to increase the strawberry fruit yield under reduced chemical fertilizers [9] or to control the photosynthetic plant's ability and N_2_O emissions under changing soil moisture levels [10]. Moreover, AMF were characterized as microbial biostimulants because of their positive contribution to plant nutrition efficiency, water balance and tolerance to biotic and abiotic stress [11]. For instance, the inoculation with arbuscular mycorrhizal fungi alleviates the acidity and aluminium toxicity in *Curcubita pepo* L. [12] or increase the macronutrient concentration of leaf tissue of a cucumber genotype under alkaline conditions [13].

For their part, essential oils and their ingredients alone induce both stimulatory and inhibitory effects on plants, herbivores, microorganisms [14],[15] and soil enzymes [16]. They may increase or decrease the size of bacterial populations, change the microbial community profiles and influence the activity of various microbial taxa [17],[18],[19]. Hassiotis [20], Hassiotis and Dina [21] found depressing effects of essential oils on AM fungi. Moreover, Papatheodorou et al. [16] reported that specific constituents of essential oils, such as R- and S-carvone, affected the profiles of soil enzymes in a selective way. Because of the above mentioned effects, plant secondary metabolites are used as alternatives for pest management [22] and especially essential oils are used as natural fungicides or bioherbicides [17],[23]. Herewith, we explored the joint effect of AMF inoculation and essential oil of *Mentha spicata* on soil microbial attributes and soil enzymatic activities. The necessity to study their joint effect comes from the fact that although both are agents of sustainable agriculture with mostly positive, independent effect on pest management and plant growth [17],[24],[25], their joint effect is largely unexplored. In a tomato pot experiment we examined the independent and combined effects exerted by the inoculation with the arbuscular-mycorrhizal fungus (AMF) *R. irregularis* and the repeated addition of essential oil extracted from *M. spicata* (spearmint) leaves, on the community profiles of free-living microbes and on soil enzymes, mainly those involved in the N-cycle since this cycle is entirely microbially-mediated.

The present study is restricted to the effects exerted during the initial pre-symbiotic phase. This period, which involves spore germination of AM fungi, hyphal growth, plant recognition and formation of appressoria is crucial for the unfolding of the whole colonization process. The plant signals that stimulate spore germination and hyphal branching are associated with root exudates and more precisely with strigolactones [26], while the growing extraradical hyphae produce mycorrhizal factors that activate symbiosis-related plant genes [27]. During this early root colonization phase, the regulatory role of various secondary plant compounds, such as flavonoid [28] or phenolic compounds [29] is highly important. The leaves and leaf extracts of spearmint (*M. spicata*) are rich in such kind of compounds; flavonoids [30],[31], monoterpenes and their derivatives [32].

Arbuscular mycorrhizal fungi interact with the members of the soil microbial community producing suppressive or stimulative effects while essential oil could be toxic to some microbes and beneficial to some others acting as food source. In this line, the hypothesis that was explored in this paper is that the joint effect of AMF inoculation and essential oil addition would be synergistic to the members of the soil microbial community resulting in higher microbial biomasses compared to values recorded in singly treated pots. Moreover, we hypothesized that these changes in the community would be reflected on the soil enzyme activities.

## 2. Materials and Method

### 2.1. Experimental design

Tomato seedlings (*Solanum* *lycopersicun*) originated from sterilized seeds were grown in hydroponic cultures for a period of three weeks and were singly transplanted into 18 experimental pots. Pots were surface sterilized (2 L volume) and filled with sterilized soil-sand mixture (1500 g in each pot at a soil:sand ratio 1:1 w/w). Soil was an acid (pH 5) sandy loam one, poor in organic carbon (C% 1.62) and nutrients (N% 0.096, Pextr 2.1 mg/100 g, K 1.62 mg/Kg, Ca 1.17 mg/Kg, Mg 0.18 mg/Kg and Na 0.136 mg/Kg). Its chemical properties ensured the successful colonization of plants with the AMF. Further, to eradicate the indigenous AMF and other soil borne biota, the soil-sand mixture was sterilized by autoclaving (4 h at 120 °C).

Before transplantation the roots of seedlings planted in nine pots were inoculated with *R. irregularis* inoculum. The root system of each seedling was sprinkled with the inoculum powder. The inoculum consisting of spores and hyphal fragments of *R. irregularis*, was provided by the Energy and Resource Institute, India and its concentration was 1000 propagules per gram. The quality of the inoculum was checked prior to the application. Nine pots without inoculation were used as controls.

Ten days after root inoculation, we reintroduced the pre-existing bacterial community of the soil, by adding a bacterial inoculum prepared from the initially collected soil. For the preparation of the bacterial inoculum, 10 g of the initially collected soil were mixed with 50 ml of deionized water, the soil suspension was filtered through a 21 µm sieve, and 10 ml of it was added to each pot near the rhizosphere zone. The 10-days delay between AMF inoculation and the addition of bacterial inoculum was necessary for plant-fungus interactions to take place [33]. These experimental practices are common in such experiments [34] since soil microbiota often reduce the extent of AMF root colonization, driving the experimental design out of the main hypothesis [35].

Six pots (three inoculated and three non-inoculated) were not further treated and were destructively sampled 30 days after the AMF inoculation as this time was sufficient for the development of the extraradical structures of *R. irregularis* (Experiment 1). The remaining 12 pots (six inoculated and six controls) were further treated as follows: half of the inoculated pots (three) along with the three non-inoculated pots were treated with *M. spicata* essential oil. The spearmint oil was supplied by Etherio, Research and Commerce, Eratera, Greece and it was pure essential oil produced after distillation of *M. spicata* plants. The major compounds of *M. spicata* oil were carvone 63.9% and limonene 13.3% followed by 1,8-cineole, β-pinene, myrcene and α-pinene in percentages 7.1, 2.8, 2.4 and 1.4%, respectively. Thus, during the experimental period, a bifactorial design with two independent factors (AMF inoculation and oil addition) was set up. The oil was added at a weekly rate of 1.33 ml per pot, for a period of one month. The quantity of essential oil was according to a scheme developed and tested earlier and repeatedly applied since [19],[36]. The experiments were conducted in a greenhouse under natural light conditions for a two-month period (from mid-June to mid-August). During the period of plant growth, the day temperature ranged from 28–37 °C and the night temperature from 20–27 °C. The plants were watered daily in order to achieve 60% of the water holding soil capacity. No fertilizers were added.

Overall, there were four treatments with three replicates per treatment giving a total of 12 pots in a randomized block design: (i) inoculated pots with oil added (+AMF, +OIL), (ii) inoculated pots without oil (+AMF, −OIL), (iii) non-inoculated pots with oil added (−AMF, +OIL) and (iv) non-inoculated pots without oil (−AMF, −OIL; control). The second destructive sampling was conducted four weeks after the first essential oil application (Experiment 2).

### 2.2 Soil sampling

At each sampling occasion we followed a destructive sampling. From each pot we collected six subsamples (2 cm diameter × 15 cm height) at a distance of 5 cm in a circle around the plant. The subsamples were mixed in order to get one composite soil sample per pot. These composite samples were analyzed for PLFAs concentration and enzyme activities. Each composite sample was sieved through a 2 mm mesh to remove roots and organic debris and from 1 mm mesh to keep sand particles away. Then samples stored at a constant temperature at 4 °C until used, within the same week.

### 2.3. AMF analysis

Plant roots were cleaned from any soil or sand particles by use of an ultrasonic bath at 50 Hz. Then, the roots were excised carefully with scissors or scalpel and forceps, as appropriate, and stored at −20 °C before use. Prior to estimating the colonization rate by AMF, all roots were put in 50% (v/v) ethanol at 5 °C. For the estimation of the AMF colonization, 40% stratified random root samples were used. Roots were rinsed with distilled water, and then stained with 0.05% (w/v) Trypan Blue in acidic glycerol. They were then incubated in 10% (v/v) KOH, at room temperature, for 24 h. The method was originally proposed by Koske and Gemma [37] and was modified by Orfanoudakis et al. [38]. The stained samples were examined under compound microscope, and the AMF percentage colonization was evaluated according to Trouvelot et al. [39]. Two slides from each sample containing 15 root fragments were mounted in glycerol. These fragments were observed under the microscope and the level of mycorrhizal colonization and abundance of arbuscules were estimated.

### 2.4. Enzyme activity assays

The activities of six soil enzymes were studied. These were *N*-acetyl-glucosaminidase (NAG), acid phosphatase, urease, asparaginase, glutaminase and arylamidase. *N*-acetyl-glucosaminidase and acid phosphatase activities were determined according to the procedures of Allison and Jastrow [40], as these were modified in order to be applicable for 96-well microplates. Approximately 1–2 g fresh soil (equivalent to 0.5 g dry weight) were added in 60 ml of 50 mM sodium acetate buffer, pH 5, and homogenized in a blender for 1 min. Then, 50 µL of homogenized soil slurry were combined with 150 µL substrate solution and incubated for 3 h (NAG) or 1 h (acid phosphatase) at 21 °C under constant shaking. Substrate solutions were 2 mM *p*-nitrophenyl-*β*-*N*-acetylglucosaminide for NAG and 5 mM *p*-nitrophenyl-phosphate for phosphatase, all in acetate buffer. After incubation, 100 mL of the slurry-substrate supernatant (without soil particles) were carefully transferred to another microplate for colorimetric determination of product concentrations. The *p*-nitrophenol (pNP) reaction product from the phosphatase and NAG assays was measured at 405 nm, after addition of sodium hydroxide. Eight replicates were run per sample; in each case, we included appropriate controls to estimate the background absorbance of the substrate and homogenate. The activity of the two enzymes is presented in units of µmol pNP g^−1^ dry soil h^−1^.

Urease activity was determined according to the methods of Sinsabaugh et al. [41]. The microplate configuration was similar to that described for the NAG assay. The concentration of urea in the assay wells was 20 mM. The plates were incubated at 20 °C for approximately 18 h. Ammonium released by the reaction was quantified using colorimetric salicylate and cyanurate reagent packages from Hach. Urease activity was measured spectrophotometrically at 610 nm. Activity is expressed as micromoles of ammonium released per hour per g soil (µmol NH_4_^+^ g^−1^ h^−1^).

The activities of asparaginase and glutaminase were determined according to the methods of Tabatabai [42]. Briefly, the methods are based on the determination of NH_4_^+^ released when soil is incubated at 37 °C for 2 h with 0.1 M tris-hydroxymethyl-aminomethane (THAM) buffer, toluene and L-asparagine or L-glutamine for asparaginase and glutaminase, respectively. The NH_4_^+^ released was determined by treating the incubated soil sample with 2 M KCl containing Ag_2_SO_4_ (to stop the enzymatic activity) followed by steam distillation of an aliquot of the resulting soil suspension with MgO. The activities of these enzymes were assayed on <2 mm field-moist samples, at the optimal pH value, in duplicates and one control, and are expressed on a moisture-free basis. Moisture was determined after weight reduction when drying at 105 °C for 24 h.

Arylamidase activity was evaluated according to the method of Acosta-Martínez and Tabatabai [43]. 1 g air-dried soil was incubated at 37 °C for 1 h with the substrate L-leucine-*β*-naphthylamide in THAM buffer (0.1 M, pH = 8.0). The reaction was stopped with ethanol and the product *β*-naphthylamide was measured colorimetrically at 540 nm after its reaction with *p*-dimethylamino-cinnamaldehyde.

### 2.5. Phospholipid fatty acid analysis

Extraction and analysis of phospholipids from soil samples was performed always within a week, as described by Papadopoulou et al. [44]. The steps are briefly the following: (i) extraction of lipids, (ii) separation of phospholipids by column chromatography, (iii) methylation of esterified fatty acids in the phospholipid fraction, (iv) chromatographic separation and identification of the main components on a Trace GC Ultra gas chromatograph (Thermo Finnigan, San Jose, CA) coupled with a Trace ISQ mass spectrometry detector, a split-splitless injector, and an Xcalibur MS platform. The column was a 5% phenyl methylsiloxane fused silica capillary column (HP-5MS, 26 m length × 0.250 mm i.d., film thickness 0.25 µm). The injector and transfer line were at 250 °C, the interface at 275 °C, and electron energy in electron impact was 70 eV. The oven temperature was programmed as follows: 120 °C (held for 7 min), then raised to 170 °C (4 °C/min) isothermally held for 3 min, and finally raised to 250 °C (3 °C/min) and held for 2 min. Helium was the carrier gas at a constant flow rate of 1 mL/min; 1 µL of each sample was injected in the splitless mode (90 s). Mass spectrometry acquisition was carried out using the continuous [electron ionization (EI) positive] scanning mode from 40 to 500 amu. Fatty acid methyl esters (FAME) were identified by (i) comparison of their relative retention times and mass fragmentation to those of authentic commercial fatty acids methyl esters standard mixtures FAME (47885-U) and BAME (47080-U) (Supelco, United Kingdom) and (ii) computer matching against a NIST98 commercial library, National Institute of Standards and Technology (NIST), Gaithersburg, MD. Quantification of each fatty acid (in nmol g^−1^) was achieved by one point calibration against the GC response of the internal standard 19:0 ME. Under the above described conditions the GC response to 19:0 methyl ester was linear in the range of 25–200 µg ml^−1^, with acceptable recoveries [45].

The total amount of PLFAs was used to account for the total microbial biomass. The fatty acid nomenclature was according to Papadopoulou et al. [44]. Overall, 21 fatty acid methyl esters were identified and considered for further analysis, including the internal standard 19:0. These are i15:0, a15:0, 15:0, i16:0, i17:0 which are indicators of Gram^+^ bacteria [46],[47],[48], the bacteria indicators 16:0, 17:0 [49], the Gram^−^ bacteria indicator 16:1ω9c [48] and the indicators of actinomycetes 10Me16:0, 10Me17:0, 10Me18:0 [50],[51]. All these are considered to be of bacterial origin only and were chosen to represent bacterial biomass. The 18:1ω9c and 18:2ω9,12 fatty acids are used as indicators of fungal biomass [48],[49], while the fatty acids 17:1, 18:0 and 14:0 are mainly of microbial origin. Finally, the PLFAs 20:0, 22:0, 23:0, 24:0 are considered as indicators of microeukaryotes (algae, protozoa, nematodes) [52]. Moreover, the ratios Gram^+^/Gram^−^, fungi/bacteria (F/B) and iso/anteiso are estimated. Iso biomass is equal to the sum of i15:0, i16:0 and i17:0 biomasses, while anteiso is represented by the biomass of a15:0.

### 2.6. Data analyses

The first data set (Experiment 1) included data gathered 30 and 60 days after inoculation (from both inoculated and non-inoculated pots). The experimental design was full factorial with inoculation and time being the independent variables, each with two levels (inoculation: Yes-No and time: 30–60 days).

The second data set (Experiment 2) included recordings 60 days after inoculation and 30 days after the first oil addition from all the combinations of inoculated and treated with oil (controls included) samples. The design was full factorial with inoculation and addition of oil being the independent variables, each with two levels (Yes-No).

Prior to analysis, for both data sets, we estimated values for skewness, which indicated asymmetry in most variables. Therefore, we applied analyses of variance (ANOVA) by using the Generalized Linear Model package (Statsoft Inc. Tulsa, USA) for completely randomized full factorial designs, each with 2 × 2 treatments (three genuine replicates per treatment). In Experiment 1, AM fungus inoculation (levels: +AMF, −AMF) and time of sampling (levels: 1 and 2) were the independent variables, with Gamma and Log being the distribution and link functions, respectively. Similarly, a two-way analysis of variance was applied on the data of Experiment 2 with AM fungus inoculation (levels: +AMF, −AMF) and addition of essential oil (levels: +OIL, −OIL) being the independent variables.

The Principal Component and Classification Analysis (PCCA) module of STATISTICA was chosen to explore further the effect of treatments on the values of enzymatic activity, PLFA of certain microbial groups, and PLFA ratios in each treatment. Due to differences in the measurement scales, data were rescaled in the range 0–1 before statistical analysis. Moreover, to correct non-normality in data distribution for actinomycetes biomass, the ratio F/B and the NAG activity, a square root for the first two variables and an inverse square root transformation for the latter were performed. The analysis was separately conducted for data from each experiment.

Finally, in order to examine whether inoculation with the AM fungus and the essential oil addition exerted differential selective pressures on the microbial community structure, values from the second data set relating to specific PLFA biomass were assessed employing PCCA. Results were depicted on the two first axes of the loading plot. Preliminary assessment did not reveal asymmetries, thus the non-transformed data set was used.

## 3. Results

### 3.1. ΑΜF inoculation

There were no signs of AMF colonization of the non-inoculated pots even sixty days after the start of the experiment. In inoculated samples, appressoria were visible and low percentage colonization was observed at both first and second sampling dates; mean colonization rate were 0.13% and 0.77%, respectively. The addition of the essential oil resulted in the decrease of the mean abundance of formed arbuscules (from 0.75 to 0.15) (Table 1). The fact that the current study took place the period preceding the switch to the symbiotic phase was supported by the data collected at 66, 76 and 90 days after inoculation in inoculated pots. In 66 days the rate of root colonization and arbuscules' formation was 48.5% and 53% respectively.

**Table 1.** Average percent colonization and abundance of arbuscules (±st. error) 30, 60, 66, 76 and 90 days after inoculation of potted tomatoes' roots with *Rhizophagous irregularis*.

Treatment	M%	a%
30d (−AMF)	0	0
30d (+AMF)	0.13 ± 0.035	0.06 ± 0.015
60d (−AMF, −OIL)	0	0
60d (−AMF, +OIL)	0	0
60d (+AMF, −OIL)	0.77 ± 0.089	0.75 ± 0.029
60d (+AMF, +OIL)	0.99 ± 0.095	0.15 ± 0.086
66d (+AMF, −OIL)	48.50 ± 9.89	53.00 ± 0.42
76d (+AMF, −OIL)	72.00 ± 1.14	92.15 ± 4.59
90d (+AMF, −OIL)	82.50 ± 4.17	90.00 ± 2.36

M%: intensity of hyphal colonization; a%: abundance of formed arbuscules; −AMF and +AMF: non-inoculated and inoculated pots respectively; +OIL and −OIL: with and without essential oil.

### 3.2. AMF effects (Experiment 1)

**Figure 1.**
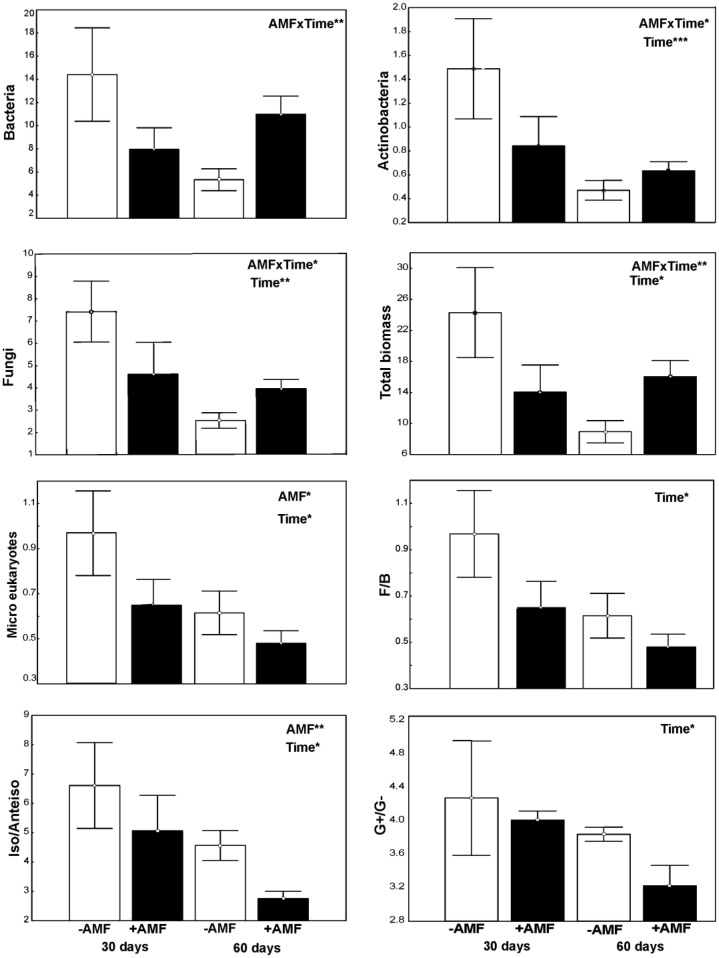
Biomass (mean ± SE, n = 3) of different microbial groups (nmols g^−1^) and PLFAs ratios in inoculated (black bars) and non-inoculated (white bars) pots, 30 and 60 days after inoculation. AMF indicates a significant effect of inoculation and Time indicates a significant effect of sampling revealed by a generalized Two-way ANOVA (Distribution: Gamma. Link function: Log). *** *P* < 0.001, ** *P* < 0.01, * *P* < 0.05.

The total microbial biomass as well as that of the various groups, with the exception of microeukaryotes, was not affected by inoculation per se, but by the combined effect of inoculation × sampling time (Figure 1). Apart from bacteria, for the rest groups the independent effect of sampling time was also significant, reflecting the effect of plant development on the biomass of free microbes.

**Figure 2.**
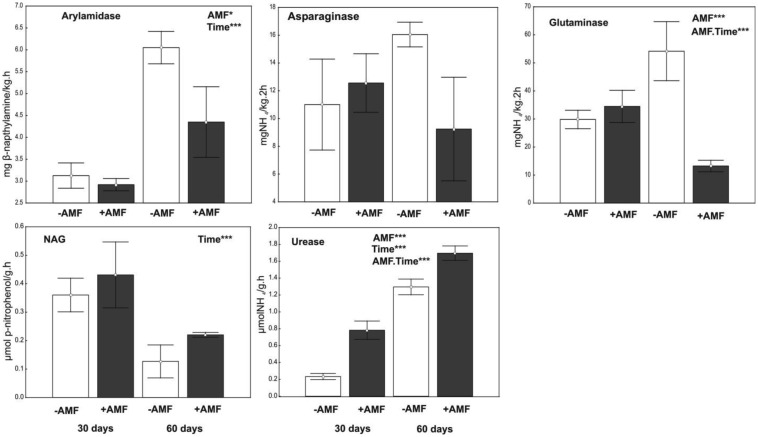
Activity (mean ± SE, n = 3) of soil enzymes in inoculated (gray bars) and non-inoculated (white bars) pots, 30 and 60 days after inoculation. AMF indicates a significant effect of inoculation and Time indicates a significant effect of sampling revealed by a generalized Two-way ANOVA (Distribution: Gamma. Link function: Log). *** *P* < 0.001, * *P* < 0.05.

On the contrary, inoculation exerted a significant effect on the activity of arylamidase, glutaminase and urease, whereas sampling time had an effect on the activity of arylamidase, NAG and urease (Figure 2). At the first sampling, the biomass of every microbial group examined was negatively affected by inoculation, whereas the activity of urease was positively affected. In contrast, at the second sampling, inoculation had a positive effect on bacteria and fungi and a negative one on micro eukaryotes. The activities of arylamidase and glutaminase were affected negatively by AMF and that of urease positively at this second sampling. Concerning the PLFAs ratios, their values were affected mainly by sampling time; they decreased from first to second sampling. Moreover, the iso/anteiso ratio was lower in inoculated pots compared to the non-inoculated ones.

**Figure 3.**
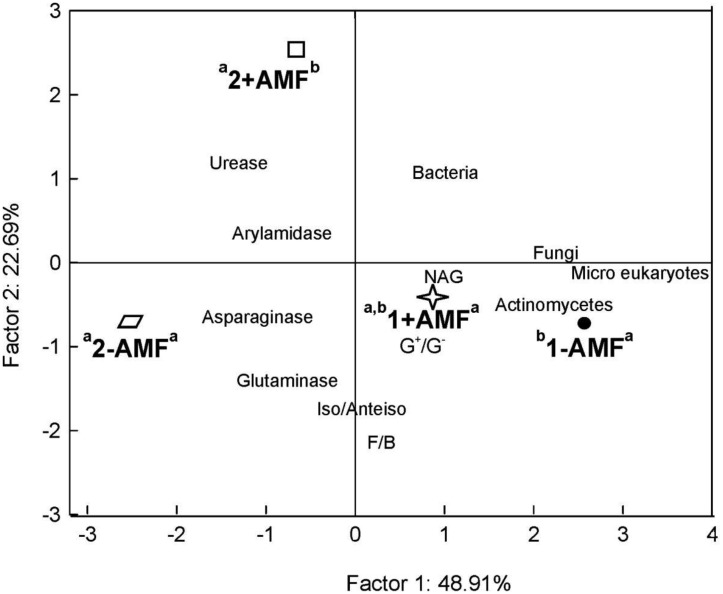
Ordination biplot diagram of Principal Component & Classification Analysis (PCCA) displaying the ordination of treatments [inoculated (+AMF) and non-inoculated samples (−AMF) at two sampling dates (1:30 d and 2:60 d after inoculation)] in relation to the biomass of microbial groups, PLFA ratios and values of enzymatic activity. Letters above labels indicate significant differences of the corresponding groups' centroids in relation to the first (left) and second (right) ordination axes.

The ordination of treatments in relation to the biomass of the various microbial l groups, PLFA ratios and enzymatic activity values, as a result of the PCCA analysis, is presented in Figure 3. One-way ANOVA applied on to the sample scores estimated for both axes revealed significant separation of treatments. Along the first axis, explaining 49% of data variability, the effect of sampling time was clearly depicted. The inoculation effect was expressed in relation to the second axis, in samples taken at the second sampling date. In fact, inoculated and non-inoculated samples from the second sampling date tended to be ordinated towards the opposite ends of the second axis, whereas the distance between their groups' centroids was significantly different from zero. In contrast, the distance between the centroids of the inoculated and non-inoculated groups taken at the first sampling date was not significantly different from zero. As shown in Figure 3, PLFA data were ordinated along with samples taken at the first sampling. In contrast, higher enzymatic activities were associated with samples from the second sampling. Specifically, high urease activity was ordinated along with inoculated samples, while high asparaginase and glutaminase activity were classified along with the non-inoculated ones. The lack of overlapping between inoculated and non-inoculated samples at the second sampling, figured on the PCCA plot, indicates unique PLFA and enzyme profiles associated with inoculation.

### 3.3. AMF and essential oil effects (Experiment 2)

**Figure 4.**
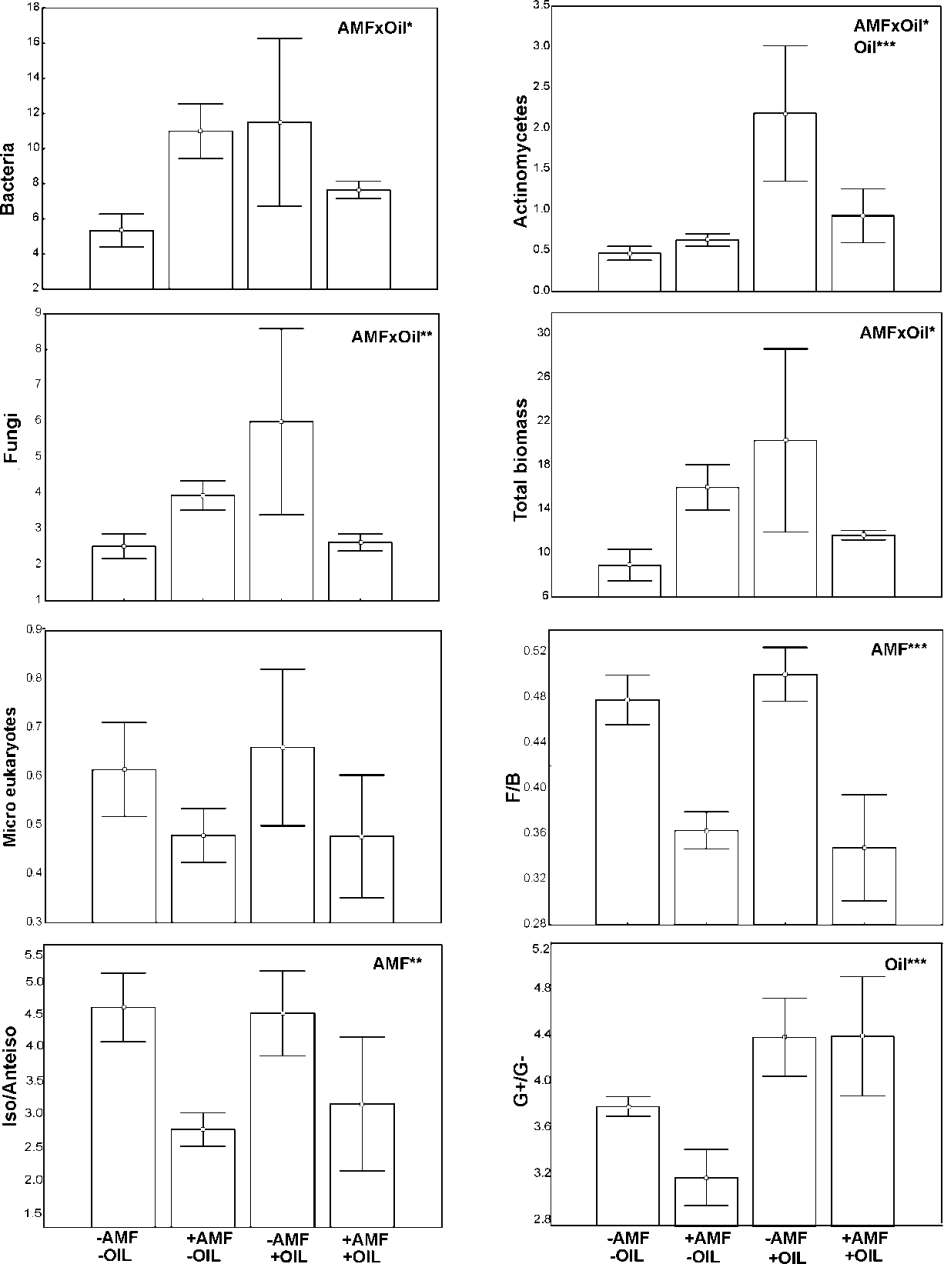
Biomass (mean ± SE, n = 3) of different microbial groups (nmols g^−1^) and PLFAs ratios in inoculated (+AMF) and non-inoculated (−AMF) with (+OIL) and without (−OIL) essential oil pots. AMF indicates a significant effect of inoculation and OIL indicates a significant effect of oil addition revealed by a generalized Two-way ANOVA (Distribution: Gamma. Link function: Log). *** *P* < 0.001, ** *P* < 0.01, * *P* < 0.05.

**Figure 5.**
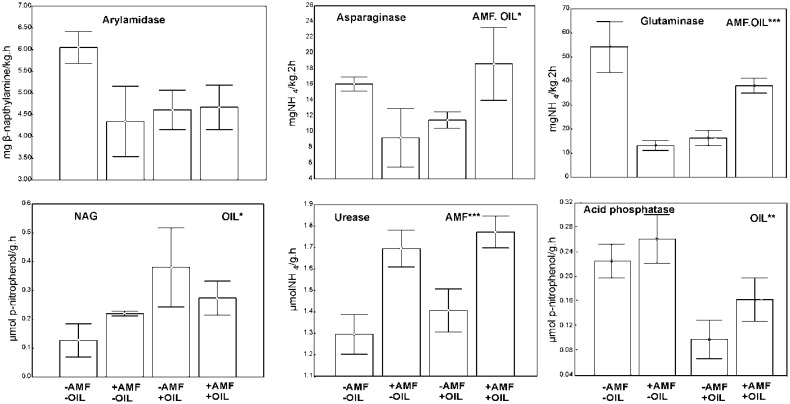
Activity (mean ± SE, n = 3) of soil enzymes in inoculated (+AMF) and non-inoculated (−AMF) with (+OIL) and without (−OIL) essential oil pots. AMF indicates a significant effect of inoculation and OIL indicates a significant effect of oil addition revealed by a generalized Two-way ANOVA (Distribution: Gamma. Link function: Log). *** *P* < 0.001, ** *P* < 0.01, * *P* < 0.05.

The biomasses of the most microbial groups were affected by the joint effect of the two treatments; inoculation and addition of essential oil (Figure 4). Lower values were recorded in control samples (−AMF, −OIL) followed by samples subjected to both treatments (+AMF, +OIL). Inoculation and essential oils affected microbial biomasses in opposite directions. Inoculation per se influenced significantly the ratios F/B and iso/anteiso biomarkers. High values of F/B and iso/anteiso ratios were recorded in the non-inoculated samples (−AMF) while the ratio Gram^+^/Cram^−^ was higher in oil treated samples. Regarding enzymatic activity, a joint effect of the two treatments was detected on asparaginase and glutaminase with the activities of both being high in the control samples and in samples subjected to both treatments. On the contrary, NAG and phosphatase activities were affected only by oil, though exhibiting a reverse pattern; NAG activity was higher in oil-treated samples while that of phosphatase in samples without oil (Figure 5).

**Figure 6.**
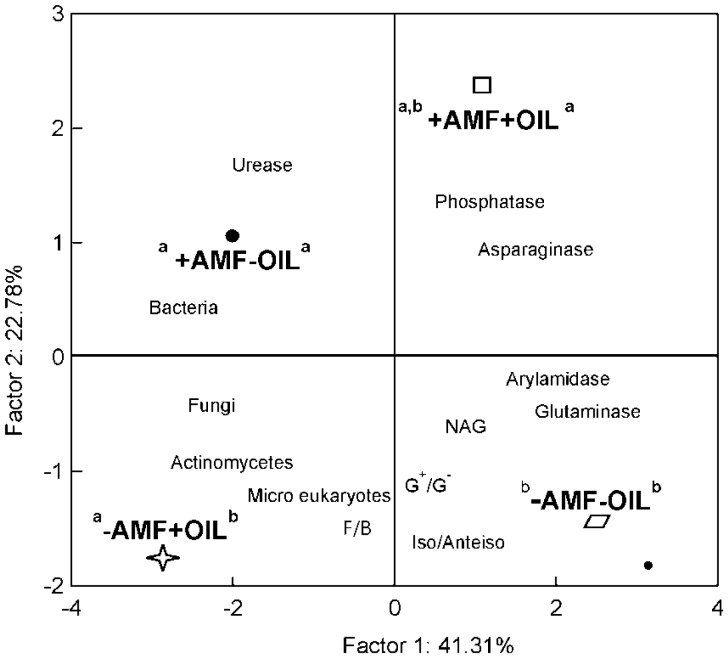
Ordination biplot diagram of Principal Component & Classification Analysis (PCCA) displaying the ordination of treatments [inoculated (+AMF) and non-inoculated samples (−AMF), with (+OIL) and without (−OIL) essential oil] in relation to the biomasses of microbial groups, PLFA ratios and values of enzymatic activity. Letters above labels indicate significant differences of the corresponding groups' centroids in relation to the first (left) and second (right) ordination axes.

Along the first axis of the PCCA loading plot, which accounted for 42% of the data variability, the essential-oil's effect was figured separately for the non-inoculated pots (lower part) and the inoculated ones (upper part), although in opposite directions (Figure 6). The inoculation effect was depicted along the second axis that accounted for 23% of data variability, with F/B, Gram^+^/Gram^−^ and iso/anteiso being associated with the non-inoculated samples. Biomass markers were negatively loaded on the first axis and pointed towards pots subjected to single treatments, either addition of oil or inoculation. On the contrary, enzymatic activity pointed either to pots subjected to both treatments or to control pots. Glutaminase and arylamidase were associated with control pots, while urease, asparaginase and phosphatase were associated with inoculated pots. Again, apart from the overlapping between +AMF, +OIL and +AMF, −OIL samples, the samples of the rest treatments were clearly distinct indicating unique PLFA and enzyme profiles.

**Figure 7.**
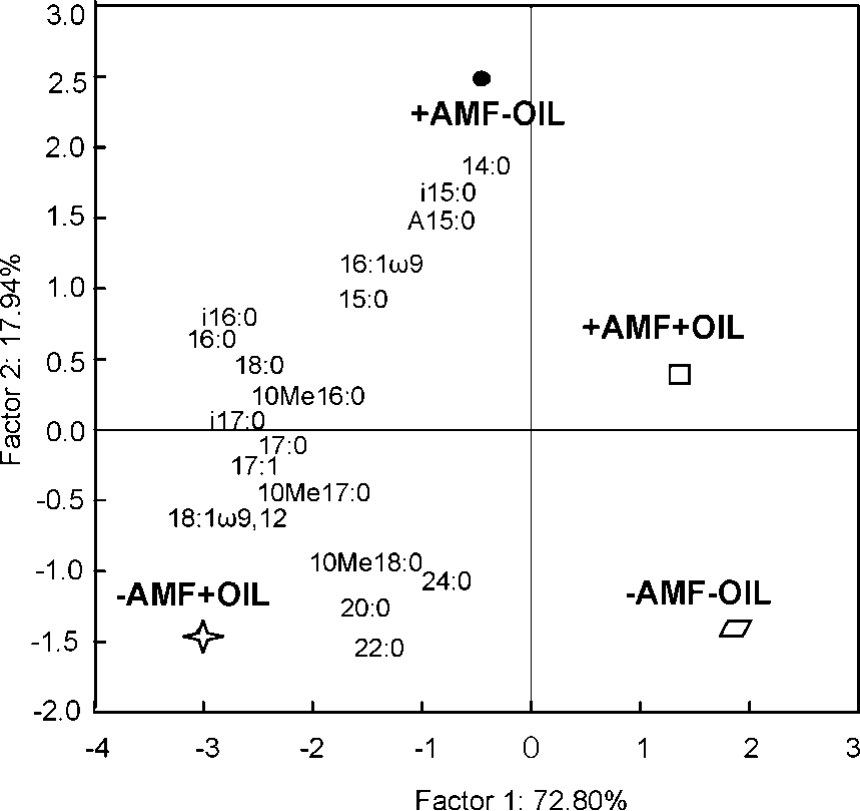
Ordination biplot diagram of Principal Component & Classification Analysis (PCCA) displaying the ordination of treatments (for details see Figure 6) in relation to the biomass of specific PLFAs on the phase plot of the first two ordination axes.

The specific PLFAs profiles were further analyzed by PCCA (Figure 7). The centroids of treatments' groups were not significantly distinguished along the first axis, whereas their discrimination along the second axis was significant (one way-ANOVA). The Gram^+^ biomarkers i15:0, a15:0, 15:0, the Gram^−^ marker 16:1ω9 and the microbial marker 14:0 were positively loaded on the second axis along with inoculated samples. In contrast, the actinomycetes marker 10Me18:0 and the micro eukaryotes markers 20:0, 22:0 and 24:0 were negatively loaded along the second axis running with non-inoculated but treated with essential oil samples.

## 4. Discussion

The colonization rates that were recorded sixty days after inoculation with *R. irregularis* were lower compared to those reported by other authors [53],[54]. Low colonization rates are usually attributed either to excess in P [26] and/or to root exudation of inhibitory compounds before the formation of appressoria [55],[56]. The hypothesis postulating exudation of inhibitory compounds was not supported since appressoria were visible at sixty days. In relation to P concentration, although its value in the sandy loam soil used for mesocosms was low, its concentration could be different after sterilization because of NH_4_^+^ release [57], which could affect rhizosphere pH and consequently P availability. Moreover, the changes in pH affect the intensity of AM root colonization [58].

### 4.1. AMF effects (Experiment 1)

Depending on the sampling time, differences in the response of microbial biomass to inoculation was revealed. After 30 days the total microbial biomass was lower in the inoculated pots compared to the non-inoculated ones, but the reverse was recorded after 60 days when mycelia started growing. Although, to the best of our knowledge, such reversals have not been reported before, there is some information in support of this phenomenon. For instance, Christensen and Jakobsen [59] reported that four weeks after inoculation of cucumber pots with AMF, the bacterial biomass was decreased, which is in agreement with our first-sampling results, while Zarea et al. [60], in a clover pot experiment, observed an increase of microbial biomass at the end of the growing period, after three months; the latter is consistent with our second-sampling results. A positive effect of AMF on the biomasses of free-living microbes was recorded by Ladygina et al. [61] after 20 weeks of inoculation and it was attributed to the positive contribution of AMF to soil aggregation. In our case, such an improvement of soil conditions was not supported because the time since inoculation was limited (8 instead of 20 weeks). The positive relations among AMF and free microbes could be due to the fact that AMF amplify the pool of plant root exudates and could provide carbon and nutrients to free-living microorganisms [25],[62]. This is further supported by the fact that in inoculated pots the difference in the size of the microbial biomass between the two samplings was non-significant, contrary to what happened in the non-inoculated pots. In these latter, as tomato plants are grown a strong competition among plants and soil microbes could be developed, decreasing the microbial biomass. We could hypothesize that at the first sampling this competition was alleviated by possible flush of nutrients in the substrate induced by sterilization [57].

The activity of enzymes showed idiosyncratic response to inoculation and time of sampling. The activity of NAG, which is involved in N- and C-cycling [63], was high in both inoculated and non-inoculated pots at the first sampling date. This can be related to the decomposition of dead microbial cells (especially fungal) resulting from the initial sterilized substrate. Through this process, nutrients became available in the experimental substrate that was poor in C and N so as to satisfy the requirements of growing roots and free living microbes. In inoculated pots, the response of NAG activity is similar to that of chitinases. The activity of the latter is triggered by signals exchanged between AM symbionts during the early stages of the colonization process [64].

Inoculation exerted a stimulating effect on the activity of urease (Figure 2). This response that is frequently reported [24],[65] is partially associated with exudations of extaradical mycelia [66],[67], structures that were not recorded herewith because the phase was the pre-symbiotic one. Increased urease activity in the inoculated pots, at the second sampling date, can function as a nitrogen providing mechanism serving the increasing needs of growing AMF [6]. On the contrary, glutaminase and arylamidase exhibited low activity in inoculated samples at the second sampling date. It appears that AMF exerted an inhibitory effect on the activity of these enzymes. However, in the case of glutaminase, the low activity in inoculated samples could be also associated with low substrate availability, since fungal spores absorb glutamine from the soil to support their germination [68]. It seems that in AMF pots, urease activity was mainly responsible for providing nitrogen, while in the non-inoculated ones the activities of arylamidase and glutaminase were dominant.

### 4.2. AMF and essential oil effects (Experiment 2)

The biomass of the various microbial groups was higher in samples subjected to single treatment, either with inoculum or essential oil, compared to control samples or to samples subjected to both treatments (Figure 4). Increase of microbial biomass with essential oils has been often reported and interpreted as resulting from the supply of a readily decomposable carbon source to microorganisms [19],[36]. Increase in NAG activity with oil addition could be attributed to the detrimental effect of oil on specific microbial strains as opposed to the beneficial effect on others [17],[69], leading to the predominance of chitinolytic bacteria [70]. Alternatively, it could be considered a response to changes in microbial stoichiometric demand, according to the microbial resource allocation theory [71]. Specifically, as essential oil provided a labile C source, microbial cells increased production of NAG to meet their demand in N.

The benefits for some microbes induced by AMF inoculation and oil addition are potentially accompanied by negative influence on other microbial strains. Inspection of the Gram^+^/Gram^−^ indicated a shifting towards predominance of Gram^+^ bacteria in oil-treated samples. Gram^+^ bacteria are known to cope with severe side effects of compounds, such as atrazine [66], which could be partly explained by their relatively thick walls and ability to form endospores [72]. Likewise, several monoterpenes, e.g. R-carvone which is the major constituent of the essential oil of *M. spicata*, are known to exert denaturing impacts to cellular membranes [73],[74]. Hence, the repeated weekly application of oil, for a period of one month, might cause damages to membranes of certain strains, while the cell wall structure may have helped other groups to overcome the severe effect of R-carvone. A shift in dominance among fungi and bacteria was declared by the inspection of the F/B ratio, and in our case was accompanied by analogous changes in the iso/anteiso ratio; both ratios increased their value in the non-inoculated pots. The higher values of the iso/anteiso ratio were considered indicators of nutrient limitation [75], and under these conditions the fungal growth seems to be mostly favored. Moreover, this nutrient limitation was recorded in pots without AMF, supporting the ideas that (a) AMF contribute positively to nutrients capture and (b) there is competition among arbuscular mycorrhizal and free living fungi for N and C [76].

From the PCCA phase graph (Figure 6), two major findings were revealed: (a) unique PLFA and enzymatic profiles for some treatments and (b) negative association of microbial biomass, particularly that of bacteria, with hydrolytic enzymes (apart from NAG activity). The first reflects unique microbial community structure and unique metabolic capacity of the communities in inoculated and non-inoculated pots at 60 days and in (−AMF, +OIL), (−AMF, −OIL) and +AMF pots independently of oil addition. This finding highlights the effect of inoculation on microbial community structure, although it is not clear herewith whether this effect is exclusively due to *R. irregularis* or to bacteria that fungal spores carried. Also, it is noticeable to mention that only in non-inoculated pots the addition of essential oil resulted in distinct microbial community composition and enzymatic profile. Unique but strong related microbial and enzyme profiles were recorded by Kaiser et al. [77], when the decomposition process was studied over the seasonal course at a beech forest.

The second finding contrasts reports demonstrating a positive correlation among the microbial biomass and the soil enzymatic activity [78]. The negative association between these two profiles could be explained by the suggestion that there is no one to one match between microbial species and enzymes; each microbial population is able to produce a relatively wide range of enzymes, whereas different microbes may produce similar enzymes [79]. Moreover, the categorization of microbes to generic groups such as G^+^, G^−^, actinomycetes etc didn't contribute positively to the identification of such a relation. Furthermore, for many enzymes, e.g. phosphatases, the activity of extracellular enzymes that are stabilized in soil colloids contributes to the estimated activity, though these specific forms are not subjected to factors regulating the current microbial activity [80].

Analysis of the specific PLFA profiles brings forth the idea of partially diverging selective powers of AM fungi and essential oil on the structure of the microbial community. Actually, in the AMF inoculated pots, the soil microbial community was characterized by three Gram^+^ and one Gram^−^ biomarkers, whereas in pots treated with oil the community was dominated by actinomycetes, that slowly mineralized relatively stable organic carbon substrates [81], and micro eukaryotes which are grazers of microbial biomass. This activity of micro eukaryotes results in enhanced rates of nutrient recycling. The biomasses of these specific PLFAs were most abundant in these singly treated samples, seemingly due to competition release. In contrast, the microbial community in samples subjected to both treatments was not characterized by particular microbial clusters, displayed a high PLFA richness and low total PLFA biomass.

## 5. Conclusion

The results of this study showed that both AMF inoculation and essential oil modulate, either independently or in concert microbial biomass, microbial community structure and enzyme activity operating as selective forces during the fungal pre-symbiotic phase. In pots treated only with essential oil, microbial community was dominated by actinomycetes and micro eukaryotes. In contrast, in pots subjected concurrently to both treatments, the diverging effects of inoculation and oil on the composition of the microbial community compensated one another and resulted to a new microbial community, suggesting non-synergistic effects of these two agents. On the contrary, a synergistic effect of the two treatments was found for asparaginase and glutaminase activity since the higher activity was recorded in pots jointly treated with oil and AMF. Therefore, we could assume that these two agents of sustainable agriculture cannot be used concurrently during the pre-symbiotic phase. In order to suggest which of them results in increased fertility, further research on the concentrations of available soil nutrients, plant development and fungal colonization process is needed.
